# Peroxisome Proliferator-Activated Receptors Regulate Hepatic Immunity and Assist in the Treatment of Primary Biliary Cholangitis

**DOI:** 10.3389/fimmu.2022.940688

**Published:** 2022-07-08

**Authors:** Chang Wang, Ying Shi, Xiaomei Wang, Heming Ma, Quan Liu, Yanhang Gao, Junqi Niu

**Affiliations:** ^1^ Department of Hepatology, The First Hospital of Jilin University, Changchun, China; ^2^ Department of Gastroenterology, Henan Provincial People’s Hospital, Zhengzhou, China; ^3^ Center of Infectious Disease and Pathogen Biology, Key Laboratory of Organ Regeneration and Transplantation of the Ministry of Education, State Key Laboratory of Zoonotic Disease, The First Hospital of Jilin University, Changchun, China; ^4^ Institute of Translational Medicine, The First Hospital of Jilin University, Changchun, China

**Keywords:** primary biliary cholangitis, fibrate, cholestatic liver disease, hepatic immunity, peroxisome proliferator-activated receptor

## Abstract

Fibrates, which are agonists of peroxisome proliferator-activated receptor alpha, have received increasing attention in the treatment of primary biliary cholangitis. Reduced alkaline phosphatase levels and improved clinical outcomes were observed in patients with primary biliary cholangitis with an inadequate response to ursodeoxycholic acid (UDCA) monotherapy4 when treated with bezafibrate or fenofibrate combined with UDCA. In contrast to obeticholic acid, which exacerbates pruritus in patients, fibrates have been shown to relieve pruritus. Clinical trial outcomes show potential for the treatment of primary biliary cholangitis by targeting peroxisome proliferator-activated receptors. It is currently agreed that primary biliary cholangitis is an autoimmune-mediated cholestatic liver disease, and peroxisome proliferator-activated receptor is a nuclear receptor that regulates the functions of multiple immune cells, thus playing an important role in regulating innate and adaptive immunity. Therefore, this review focuses on the immune disorder of primary biliary cholangitis and summarizes the regulation of hepatic immunity when peroxisome proliferator-activated receptors are targeted for treating primary biliary cholangitis.

## Introduction

Primary biliary cholangitis (PBC) is an autoimmune-mediated cholestatic liver disease characterized by progressive destruction of hepatic interlobular bile ducts, which eventually leads to liver cirrhosis (1). The diagnosis of PBC depends on elevated alkaline phosphatase (ALP)/γ-glutamyl transpeptidase (γ-GT) levels; exclusion of other diseases that may cause cholestasis, including drug-induced liver injury, biliary stones, and malignant tumors through patient medical history and imaging examinations; and positive antimitochondrial antibody (AMA) and/or antinuclear antibodies (ANA) tests, including anti-gp210 and anti-sp100. Liver biopsy may be performed when the diagnostic results for these tests are insufficient to determine PBC (2). PBC is the only cholestatic disease which has an established treatment available. Ursodeoxycholic acid (UDCA) has been the only approved treatment for PBC for over 20 years, until 2016, when obeticholic acid (OCA) was licensed for the treatment of primary biliary cholangitis (previously primary biliary cirrhosis) in combination with UDCA in adults showing an inadequate response to UDCA or as monotherapy in adults unable to tolerate UDCA (3). However, there remains a need to develop improved PBC treatments. Up to 40% of patients with PBC respond inadequately to UDCA therapy (4), and OCA aggravates pruritus dose-dependently (5, 6). Recent clinical trials of selective peroxisome proliferator-activated receptor α (SPPARα) agonists for the treatment of PBC have received increasing attention.

PPARs are ligand dependent transcription factors and three isoforms including PPARα (NR1C1), PPARβ/δ (NR1C2) and PPARγ (NR1C3) are found (7). PPAR isoforms heterodimerize with retinoid X receptor. This complex regulates gene expression by binding to specific peroxisome proliferator response elements located in regulatory site of each gene. Due to differences in tissue distribution, ligands sensitivity and target genes, these three PPAR isoforms have distinct but complementary physiological functions (8). PPARα is highly expressed in the liver, skeletal muscle, and mainly regulate lipid and glucose metabolism (9). Fibrates, which are PPARα agonists, are used to treat hyperlipidemia. PPARγ is predominantly expressed in adipose tissue, which plays an important role in insulin sensitivity (10). Thiazolidinediones, the PPARγ agonists, are used to manage type 2 diabetes. PPARβ/δ is ubiquitously expressed and is involved in many physiological processes, including lipid metabolism, wound healing and inflammation (11). PPARβ/δ agonists currently are not approved for clinical use.

The first evidence that fibrates could be used to treat hepatobiliary disease was presented in 1993, when bezafibrate treatment caused a reduction in serum and biliary ALP activities (12). In 1999– 2003, several studies from Japan attempted to treat primary biliary cirrhosis with bezafibrate (13–18), and the results of these preliminary studies indicated that bezafibrate was effective in reducing ALP, γ-GT, and immunoglobulin M (IgM), with or without UDCA. In 2002 and 2004, preliminary clinical trials of fenofibrate in the treatment of primary biliary cirrhosis were conducted in Japan, and the results suggested that combination therapy with UDCA and fenofibrate was useful in reducing ALP, γ-GT, and IgM levels (19, 20). Subsequent clinical trials and retrospective studies have provided new evidence for the use of PPAR agonists in the treatment of PBC. **Table 1** summarizes the studies that have been published and registered on the National Institutes of Health clinical trials website (http://clinicaltrials.gov). Systematic reviews and meta-analyses of these clinical trials have confirmed the efficacy of bezafibrate and fenofibrate in improving serological responses and relieving pruritus in patients (58–63). Clinical trials have attempted to treat refractory PBC with triple therapy (UDCA, OCA, and fibrate) (64, 65) with higher risks of adverse events despite a significant reduction in serological markers. Almost all clinical trials take the serological response of patients as the endpoint and lack the detection of immune-related indicators. This is due to a lack of reliable immune-related markers associated with PBC progression and prognosis. One study showed that IgM shows potential as a marker to predict the long-term clinical outcomes of patients with PBC treated with UDCA and bezafibrate (34), but further evidence is needed to confirm this assumption. In a particular clinical trial (NCT02931513), researchers evaluated whether soluble mannose receptor and soluble CD163 (sCD163), a macrophage activation marker, can be used as potential predictors of non-response to UDCA treatment and thus, as predictors of patients needing add-on therapy. Another clinical trial (NCT04514965, in progress) attempts to investigate how treatment with bezafibrate as an add-to therapy to UDCA influences the levels of sCD163, fibrosis markers, and bile acid composition in patients with PBC.

**Table 1:** clinical studies of PPAR agonists on the treatment of PBC.

Drug	UDCA combination	Number of patients	Administration time	Results	Re
**Bezafibrate** **(BZ)**	No	22	6m	21 showed a significant reduction in ALP and γ-GTP levels and IgM levels of 17 patients decreased after 6m.	(21)
	Yes/no	12/20	52w	1. BZ monotherapy was as effective as UDCA;2. BZ combined with UDCA reduced ALP in PBC patients refractory to UDCA	(22)
	Yes	15	24m	80% patients refractory to UDCA achieved normal ALP and IgM within 12m	(23)
	Yes	19	3m	ALT, AST, ALP, GGT, IgM, cholesterol, triglyceride significantly reduced	(24)
	Yes	28	1y	ALP, GGT, cholesterol and triglyceride reduced, pruritus improved and lower liver stiffness.	(25)
	Yes	13	8y	ALP and Mayo risk score were lower, creatinine was higher than UDCA monotherapy, side effects included muscle pain and renal dysfunction	(26)
	Yes	1121	6.1 ± 3.4y	Bezafibrate improve biochemical response and long-term outcome in asymptomatic patient refractory to UDCA	(27)
	No	84(include PSC)	21d	Bezafibrate was effective to treat cholestatic pruritus	(28)
	Yes	50	24m	Normal ALP in 67% patients; pruritus, fatigue, and liver stiffness were improved	(29)
	Yes	48	38m	54% patients had normalized ALP and lower jaundice, pruritus and liver stiffness	(30)
	Yes	50	24m	Pruritis was relieved	(31)
	Yes	29	48m	ALP normalization was higher and cirrhosis risk was lower.	(32)
	Yes	118	>1y	BF plus UDCA improved GLOBE and UK-PBC scores and long-term prognosis	(33)
	Yes	150	>15y	ALP, γ-GT, IgM normalization rates were higher; normalization of IgM was a good predictor of long-term prognosis	(34)
	Yes	960	40y	Bezafibrate combination therapy reduces mortality and the need for liver transplantation	(35)
	No	24	21d	Bezafibrate reduced ALP and relieved pruritus	(36)
	Yes	59	5y	Regression of fibrosis was attained in 48% of patients, and combination therapy decreased inflammatory histological scores	(37)
	Yes	746	>1y	Addition of BZ to UDCA was associated with improved transplant-free survival	(38)
	No			NCT05239468, recruiting	
	Yes			NCT04751188, recruiting	
	No			NCT04594694, recruiting	
	Yes			NCT02937012, recruiting	
	No			NCT02701166, recruiting	
**Fenofibrate**	Yes	6	8w	ALP, γ-GT, ALT, cholesterol, triglyceride significantly reduced	(39)
	Yes	20	48w	ALP, ALT, IgM, IL-1, IL-6 significantly reduced	(40)
	Yes	22	Mean 7.23m	68% of patients reached normal ALP level; γ-GT, ALT, AST significantly reduced	(41)
	Yes	14	48w	ALP, γ-GT, and IgM significantly reduced	(42)
	Yes	46	11m	Fenofibrate was associated with ALP reduction, decompensation-free and transplant-free survival in PBC patient refractory to UDCA.	(43)
	Yes	17	12m	Long-term fenofibrate treatment improves ALP level but not UK-PBC risk score	(44)
	Yes	26	>1y	Fenofibrate add-on therapy could improve ALP and γ-GT, but not UK-PBC risk and GLOBE score	(45)
	Yes	12	5-64m	Addition of fenofibrate significantly reduced ALP, ALT and AST levels	(46)
	Yes	44	3y	Fenofibrate add-on therapy improves GLOBE, UK-PBC scores, liver fibrosis and ductular injury of liver	(47)
	Yes			NCT02823353, recruiting	
	Yes			NCT02823366, recruiting	
**Pemafibrate**	Yes	7	3m	ALP, γ-GT reduced; serum plasma lipid, ALT, AST and liver fibrosis marker had no difference	(48)
	Yes	16	48w	ALP, GGT and IgM decreased significantly; pemafibrate had beneficial effects on renal function	(49)
	Yes	75	3m	Pemafibrate was efficient in reducing ALP and GGT and in improving eGFR and Cr	(50)
**Elafibranor**	No	30	12w	Elafibranor was safe and tolerated and significantly reduced ALP, bilirubin.	(51)
	No			NCT04526665, recruiting	
**Saroglitazar**	Yes	17	16w	Saroglitazar significantly reduced ALP with 50% decrease	(52)
	Yes	7	16w	Rapid and sustained improvement in ALP was observed	(53)
	No			NCT05133336, recruiting	
**Seladelpar**	Yes	23	12w	ALP levels were normalized in patients who completed 12 weeks of treatment	(54)
	Yes	101	1y	Seladelpar treatment improved pruritus, fatigue, and sleep disturbance in PBC patients	(55)
	Yes	112	6m	Seladelpar was effective in reducing ALP and pruritus	(56)
	Yes	60	52w	Seladelpar was effective in reducing ALP and pruritus	(57)
	No			NCT04620733, recruiting	
	No			NCT03301506, recruiting	
	No			NCT04950764, recruiting	

m, months; w, weeks; y, years.

Innate and adaptive immune-response abnormalities play an essential role in the occurrence and progression of PBC. Whereas PPAR, an important component of nuclear receptors, regulates the function of multiple innate and adaptive immunity-response cells. However, research on immune regulation related to PPAR in PBC is limited. Previous studies were evaluated for the regulation of PPAR on hepatic immunity in the progression of PBC to identify potentially novel biomarkers and therapeutic drugs that can be further investigated in future studies.

## Regulation of Immune Response Abnormality by PPARs in PBC

Genetic susceptibility and exposure to environmental factors are the two main contributors to PBC development. Genome-wide association studies and observations of identical twins have confirmed genetic associations and risk factors for PBC (66–68). Molecular mimicry induced by bacterial infection, especially the pyruvate dehydrogenase complex-E2 (PDC- E2), and xenobiotic exposure are important environmental factors that disrupt hepatic immune tolerance and induce PBC (69, 70). T help 1(Th1)-mediated immunopathological damage to the intrahepatic small bile duct is a characteristic of PBC (71). In fact, innate and adaptive immune-response cells collectively participate in the development of PBC at different stages of the disease, including monocytes and macrophages with hyperreaction, dendritic cells with enhanced antigen presentation, and natural killer (NK)/natural killer T (NKT) cells with enhanced killing properties in the early stage of PBC. Th17 were shown to inhibit Th2/Treg, and B cells were also involved in PBC progression. We describe the specific role of individual immune response cells in PBC progression and the regulatory role of PPARs in these cells.

### Th1/Th2

Interleukin-12 (IL-12)-induced Th1 cells produce IFN-γ and IL-2, whereas IL-4 and IL-2-induced Th2 cells secrete a variety of cytokines, including IL-4, IL-10, and IL-13 (66). Excessive Th1 immune response leads to uncontrolled tissue damage. High levels of IFNγ are associated with portal inflammation activity indicating Th1-dominant liver injury in PBC (72). Decreased IL-4 producing CD4^+^ T cells in patients with advanced PBC also supports this result (73). In addition, a trans-ethnic genome-wide meta-analysis revealed that IL12RB1 is included in the susceptibility loci of PBC, and Th1 differentiation is significant in pathway analysis (66). A decrease in liver-infiltrating CD4^+^ Th1 cells in patients with PBC indicated an adequate response to UDCA treatment (74). Therefore, reversal of the excessive Th1 immune response is of significance for the treatment of PBC.

Fibrate treatment has also been found to reduce CD4^+^ T cell migration to the liver. Bezafibrate and fenofibrate have been shown to decrease elevated normal T-cell expressed and secreted (RANTES) levels induced by chenodeoxycholic acid (75). RANTES, a member of the CC chemokine family, mediates the migration of CD4^+^ T cells to inflamed tissues, and in PBC RANTES expression has been observed to be elevated (75, 76). Research in PBC animal models also indicated that 15d-PGJ2, a PPARγ ligand, effectively attenuated portal inflammation with reduced T cell numbers, which prevented the progression of PBC (77). However, this study did not confirm the reduction in CD4^+^ T cells because of the limitation of mouse anti-CD4 antibodies.

PPARs activation also promotes Th1/Th2 phenotypic conversion, except for the inhibition of CD4^+^ T cell migration. Sex differences were found in the expression of PPARα in CD4^+^ T cells. CD4^+^ T cells isolated from female peripheral blood produced higher levels of IFNγ than those isolated from male peripheral blood. Knockdown of PPARα by small interfering RNA in male CD4^+^ T cells contributes to increased IFNγ production (78). Another study indicated that higher PPARα expression was detected in male CD4^+^ T cells than in females, and deletion of PPARα in male T cells induced increased IFNγ and TNF production (79). One study suggested a possible regulatory mechanism of PPARα in IFNγ production. Interaction of PPARα and nuclear receptor corepressor 1 reduced histone acetylation of sites on cis-regulatory elements in the ifng locus, thereby inhibiting IFNγ production and Th1 dominant immunity (80). Female predisposition characterizes multiple autoimmune diseases, including PBC. Several hypotheses, such as sex hormones, genes, and epigenetic regulation, have attempted to explain the predominance of PBC in females (81), but the reasons remain unclear. The potential relationship between differences in PPARα expression in CD4^+^ T cells and characteristics of autoimmune diseases remains to be investigated. PPARγ activation also contributes to Th1/Th2 phenotypic conversion (82, 83). Another study indicated that PPARγ binds directly to prospero-related homeobox and inhibits the production of IFNγ (84). PPARδ was also demonstrated to inhibit IFNγ production in other Th1-mediated autoimmune disease (85, 86), but these results need to be confirmed in studies on PBC.

### Th17

Th17 cells differentiate from naïve T cells stimulated by IL-1β, IL-6, and TGF-β1 and are characterized by IL-17 production. IL-23 is required to maintain Th17 cellular function (87). Recent studies have confirmed that Th17 cells play an important role in the progression of PBC, although the mechanism has not been fully elucidated. The frequency of Th17 cells in the liver tissues of patients is higher than that in healthy controls (88). Th1 and Th17 differentiation was included in the pathway analysis of the trans-ethnic genome-wide meta-analysis of PBC cohorts. Elevated IL-17 produced by Th17 cells in the liver promotes the proliferation and fibrosis of hepatic stellate cells in PBC (89).

PPARα and PPARγ are involved in the suppression of Th17 differentiation from naïve T cells by inhibiting the expression of the retinoic acid receptor-related orphan receptor (RORγt), an important factor controlling Th17 polarization (90). Fenofibrate inhibits Th17 differentiation through the IL-6/STAT3/RORγt pathway, and this effect could be reversed by MK886, a PPARα antagonist (91). Upregulation of PPARγ also selectively inhibits Th17 differentiation, but not Th1, Th2, or Treg differentiation in CD4^+^ T cells *via* inhibition of RORγt (92). PPARγ agonist has also been reported to inhibit Th17 polarization by regulating the expression of cyclin B1 and glutaminase (93; 94). Increased IFNγ and IL-17 levels have been observed in PPARδ-deficient mice, indicating an enhanced Th1/Th17 mediated immune response (95). However, a PPARδ agonist blocks IL-17 production by inhibiting Th17 function (85). These results were obtained from studies on isolated Th17 cells or other autoimmune diseases. More evidence is needed on the effects of PPAR agonists on Th17 cells after PBC treatment.

### Dendritic Cell

Dendritic cells (DC) play an essential role in the induction of an adaptive immune response. DCs from patients with PBC have a higher capacity for antigen presentation, and the presence of DCs, especially myeloid DCs, has been confirmed immunohistochemically around the damaged bile ducts (96–98). Bile epithelial cells produce macrophage protein-3α in response to IL-1β, TNFα, and IL-17, which promotes DC infiltration (99). The production of nitric oxide by DCs, which may participate in bile duct injury, was significantly higher in patients with PBC than in healthy controls (100, 101). Cytokines produced by DCs partly determine helper T cell differentiation from naïve T-cells. A study on DC subtypes found that type 2 DCs in patients with PBC were significantly decreased, which is characterized by the expression of CD123 and the promotion of Th2 cell differentiation (102). Therefore, antigen presentation and cytokines of DCs are involved in directing the Th cell response in PBC patients.

PPARα, PPARβ, and PPARγ mRNAs were detected in DCs, but only PPARγ was detected at the protein level (103). Therefore, PPARγ has been extensively studied for its role in the regulation of DC function. Isolation and culture of DCs from peripheral blood of patients with PBC indicated that bezafibrate treatment significantly decreased nitrite production in DCs (104), which was elevated in patients with PBC (100). In monocyte-induced DCs, PPARγ activation reduces DC immunogenicity and increases self-tolerance maintenance by downregulating RelB protein expression (105). Troglitazone and 15d-PGJ2, which are PPARγ ligands, inhibit toll-like receptor-mediated activation of DCs *via* inhibition of the NF-κB mitogen-activated protein kinase pathway (106). Another study indicated that troglitazone inhibited dectin-1-mediated activation by interfering with curdlan-mediated accumulation of caspase recruitment domain 9, mitogen-activated protein kinase, and the NF-κB pathway (107). Therefore, the upregulation of PPARγ may suppress the immune response in PBC by inhibiting antigen presentation by DCs.

Additionally, PPARγ activation in DCs also inhibits the Th1-dominant immune response *via* the alteration of cytokines. Activation of PPARγ in DCs maintains the immature status of DCs, which fails to promote the activation and differentiation of CD4^+^ T cells (108). Rosiglitazone, a PPARγ agonist, can downregulate CD40-induced secretion of IL-12 in DCs, a potent Th1 driving factor (109). Another study also found that PPARγ activation reduces the production of IL-12 in CD1a- monocyte-derived DCs (110). These results were confirmed in another study with unaffected production of IL-1β, IL-6, IL-10, and TNF-α. Reduced Th1 recruiting chemokines, including CXCL10 and CCL5, but not Th2-attracting chemokines including CCL22 and CCL17, were observed in this study (111). It was found through subsequent research that PPARγ directly binds to the PPAR response element in the human IL-10 promoter region, upregulating IL-10 expression of DCs (112).

### Treg

Regulatory T (Treg) cells are important in the maintenance of immune tolerance, and the forkhead transcription factor Foxp3 has been shown to be an essential regulator of Treg lineage commitment and function (113). Two subtypes of Tregs, thymus-derived natural Tregs and inducible Tregs from CD4^+^CD25^-^ T effector cells, have been described (114, 115). The suppressive effects of Treg cells from patients with PBC decreased and differentiated into Th1 cells upon stimulation with low concentrations of IL-12 (116). The relative number of CD4^+^CD25^+^ regulatory T cells and Foxp3-expressing Tregs in patients with PBC was significantly reduced compared to that in healthy controls, and the CD8^+^/Foxp3^+^ Treg ratio was markedly higher in late-stage patients with PBC than in those with chronic hepatitis C and autoimmune hepatitis (117).

*In vitro* and *in vivo* studies confirmed the regulatory effects of PPARs on Tregs. Fenofibrate promotes Foxp3^+^ regulatory T cell differentiation *in vitro* by inhibiting Akt and enhancing Smad3 phosphorylation (118). Another study found that the suppressive effect of PPARα-deficient Treg cells on CD4^+^CD25^-^ and CD8^+^ T cell proliferation was impaired (119). Bezafibrate and ciglitazone induces stable Foxp3 expression by collaborating with transforming growth factor-β through the downregulation of DNA methyltransferase, which mediates demethylation of Foxp3-conserved noncoding DNA elements (120). Pioglitazone also promotes Foxp3 expression, increasing the percentage of hepatic CD4^+^CD25^+^Foxp3^+^ Treg cells significantly (121). It seems that the upregulation of PPARs contributes to the maintenance of the inhibitory effects and high frequency of Treg cells.

### Follicular Helper T Cell

Follicular helper T (Tfh) cells are a subset of CD4^+^ T cells with the characteristic CXCR5^+^, whose main function is to regulate humoral immunity. The activation, proliferation, and differentiation to antibody-producing plasma cells depend on Tfh cells (122, 123). It has been demonstrated that the frequency of circulating CD4^+^CXCR5^+^ Tfh cells in patients with PBC is significantly higher than that in healthy controls and patients with autoimmune hepatitis (124). In this study, the frequency of Tfh cells was reduced in patients with PBC with an adequate response to UDCA treatment compared to those who showed an inadequate response to UDCA. Another study indicated that elevated Tfh cells were positively correlated with increased plasma B cells, serum AMA, and IgM in patients with PBC (125). Mice with a specific knockout of PPARγ in CD4^+^ T cells developed an autoimmune phenotype with increased activation of Tfh cells and enhanced autoantibody production of B cells. However, pioglitazone treatment significantly ameliorated the Tfh cell response (126, 127). These results confirm the regulatory effects of PPAR on Tfh cells.

### B Cell

The presence of autoantibodies and hyperglobulinemia, particularly IgM, is characteristic of PBC. IgM-producing plasma cells are significantly increased in the serum of patients with PBC (128). Presentation of PDC by cross-reactive B cells may be responsible for the disruption of T cell tolerance to highly conserved self-antigen PDC (129). Whether the levels of autoantibodies and IgM are correlated with the clinical manifestations and outcomes of patients with PBC is still controversial (130, 131). However, decreased IgM levels were observed in patients who responded adequately to UDCA monotherapy or UDCA combined with OCA or fibrates in some clinical trials. One study reported that serum IgM has the potential to be a marker for predicting long-term clinical outcomes of patients with PBC treated with UDCA and bezafibrate (34). The mechanism underlying the high levels of IgM and its role in PBC remains unclear. Genomic and miRNA analyses have indicated that IFNγ and CD40L are central upstream regulators of PBC (132). One study also found that reduced methylation of the CD40L promoter in CD4^+^ T cells was inversely associated with IgM levels in PBC (133). This provides an epigenetic regulatory explanation for the elevated IgM levels. The depletion of B cells is an immune-related treatment strategy for PBC. Decreased ALP and IgM levels were observed in patients with PBC treated with rituximab, with increased frequency of CD25highCD4^+^ T cells and increased expression of FoxP3 (134). Although B cell depletion is effective in reducing AMA and IgM levels, serological responses of patients with PBC are not always reproducible in all clinical trials (135).

The regulatory role of PPARs in B cells is limited. B cell-activating factor (BAFF) belongs to the tumor necrosis factor family, which plays an important role in B cell maturation. Overexpression of BAFF is harmful to the immune tolerance of B cells, and increased BAFF is detected in patients with PBC (136). BAFF-activated B cell-mediated Treg cell apoptosis also contributes to impaired immune tolerance, and bezafibrate treatment effectively inhibits BAFF-induced Treg apoptosis (137). In addition, in PPARγ haplo-deficient mice, the proliferation and antigen-specific immune response of B cells are upregulated (138). Research on B cell-specific PPARγ knockout mice demonstrated that reduction of IL-10 producing CD5^+^CD1dhi regulatory B cells was responsible for exaggerated hypersensitivity (139). PPAR agonist treatment has reduced IgM levels in patients with PBC in numerous clinical trials (**Table 1**), which may confirm the inhibitory effects of PPARs on B cells.

### Macrophage and Monocytes

Resident Kupffer cells and monocyte-derived macrophages from the peripheral blood comprise the hepatic macrophage population, which participates in hepatic inflammation and immune response regulation. Macrophages are roughly divided into classically or alternatively activated phenotypes, which are also called M1 or M2 phenotypes with pro-inflammatory and anti-inflammatory effects, respectively (140). Early research has found that the number of hepatic Kupffer cells is increased in patients with PBC (141). In addition, monocyte chemotactic proteins (MCP), CXCL12, and CX3CL1 in the liver tissue of patients with PBC are significantly increased, which promotes the accumulation of monocytes in the liver (142–144). Monocytes from patients with PBC exhibit higher TLR4 expression, are more sensitive to LPS stimulation, and increase the production of TNFα, IL-1β, IL-6, and IL-8 (145–147). Monocytes and macrophages also influence NK cell function and T-cell differentiation. Increased circulating CD14^low^CD16^+^ monocytes in PBC promote Th1 cell skewing and accelerate liver injury (148). Kupffer cells promote NK cell activation *via* the direct interaction between NK group 2, member D, and retinoic acid early inducible-1, with increased production of IL-12, TNFα, and IFNγ, which synergistically induces hepatic inflammation in PBC (149). A study of macrophage activation markers demonstrated that an increase in soluble CD163 and mannose receptors is consistent with an increase in ALP, and these can be used as markers to predict disease severity and prognosis of patients with PBC (150).

Previous studies have shown that PPARγ, PPARβ/δ, and PPARα exert regulatory effects on macrophages. Activation of PPARγ in Kupffer cells significantly inhibits the production of nitric oxide and TNFα, resulting in the suppression of inflammation (151). Another study showed that pioglitazone prevents LPS-induced liver injury by inhibiting TNFα production in Kupffer cells (152). Recruitment of monocytes/macrophages is reduced in cholestatic mice treated with 15d-PGJ2 (153). Several studies have confirmed that PPARδ (154) and PPARγ (155–158) activation promotes M2 macrophage polarization, which effectively inhibits hepatic inflammation. However, the mechanisms by which PPARγ and PPARδ inhibits inflammation in classically activated macrophages are distinct. SUMOylated PPARγ inhibits macrophage inflammatory gene expression by blocking the release of the nuclear receptor corepressor complex, and when PPARδ is linked to its ligands, the release of B-cell lymphoma 6 allows it to repress the transcription of inflammatory genes (159). Overexpression or absence of PPARα indicates that PPARα might also promote macrophage polarization from M1 to M2 (160, 161). Macrophages also act as intermediaries for IL-4 to suppress the secretion of IL-2 by T cells because of 12/15-lipoxygenase production in macrophages, whose metabolic product, 13-hydroxyoctadecadienoic acid, is the ligand of PPARγ (162).

### Natural Killer Cell

NK cells participate in the innate immune system, mainly through cytotoxic mechanism activation and IFNγ production. Early studies have demonstrated that the number of circulating and liver-infiltrating NK cells is significantly elevated in patients with PBC (163, 164). These increased NK cells had different properties compared with healthy controls, with increased cytotoxic activity and perforin production, but significantly decreased IFN-γ, IL-6, and IL-8 synthesis (164). Recently NK cells of patients with PBC were found to have increased sensitivity to IL-12 stimulation. A minimal amount of IL-12 stimulation can enhance IFN-γ production in NK cells (165).

PPARγ regulates NK cell cytotoxicity and IFN-γ production by interacting with PPARγ ligands. A study found that 15d-PGJ2, a natural ligand of PPARγ, simultaneously inhibited cytotoxicity and IFNγ production in NK cells, regardless of the presence of PPARγ. However, ciglitazone, a synthetic ligand of PPARγ, reduces IFNγ production *via* PPARγ activation (166).

### Natural Killer T Cell

NKT cells are lymphocytes characterized by the simultaneous expression of T-cell receptors and NK cell-related markers (CD56, CD57, and CD161) (167). In the liver, NKT cells reside in hepatic sinuses and secrete a variety of cytokines, including IFN, IL-2, IL-4, and IL-17, to induce Th1, Th2, and Th17 differentiation (168). Studies have demonstrated that NKT cells are involved in immunopathological damage in PBC. The hepatic infiltration of CD1d-αGalCer-restricted NKT cells was significantly higher in patients with PBC than in healthy controls (169). In the dnTGFβRII mouse model of PBC, the lack of CD1d-restricted NKT cells significantly decreased hepatic injury (170). Another study found that the activation of iNKT cells *via* αGalCer exacerbated hepatic damage, increased AMA production and CD8+ T cell infiltration in 2-OA-BSA, which induced PBC in the animal model (171). One study found that CD57+CD3+NKT accumulation around damaged interlobular bile ducts might be related to an imbalance in Th1/Th2 cytokines (167). CD56+and Fas ligand-positive NKT cells are involved in the death of bile epithelial cells (BECs), which promotes PBC progression. Therefore, the activation of NKT cells promotes the progression of PBC, and inhibition of NKT cells may be a potential therapeutic target for PBC.

There is no direct evidence on whether PPARs regulate NKT cells, and thus affect PBC progression. Studies on other autoimmune diseases and liver inflammation-related diseases have confirmed that PPARα and PPARγ have regulatory effects on NKT cells. One study reported that PPARα activation negatively regulates Ifng gene transcription in NKT cells, whereas PPARα antagonist enhances IFNγ production and induces Th1 dominant immunity (80). Elafibranor, a dual PPARα/δ agonist, ameliorates hepatic inflammation by reducing a variety of immune response cells, including NKT cells (172). PPARγ activation also indirectly enhances iNKT cell expansion *via* upregulation of CD1d and cathepsin D expression in DCs (89, 173). These two studies only focused on antigen presentation between DC and iNKT cells without evaluating the effects on the Th1/Th2 balance. Another study showed that iNKT cell activation enabled a Th2-dominant immune response upon PPARγ activation (174). Further research is needed to evaluate whether fibrates have the same effects on PBC treatment.

### Bile Epithelial Cell

Th-1 mediated damage to hepatic small bile ducts is characteristic of PBC, but bile epithelial cells (BECs) are not just innocent victims. BECs are involved in the maintenance of immune tolerance and immune cells including macrophages are associated with the repair of damaged BECs (175). Bacterial components recognized as pathogen-associated molecular patterns (PAMPs) are detected in bile from patients with PBC and the healthy controls (176). TLR4 in BECs is markedly expressed in patients with PBC and recognizes lipopolysaccharide (177). TLR4 interacts with the adaptor protein myeloid differentiation primary response 88 (MyD88), which recruits IL-1 receptor-associated kinase (IRAK) 1 and subsequently activates the NF-κB and MAPK signaling pathways. Owing to the activation of these pathways, BECs produce more IL-6, IL-8, and MCP-1 (178). PPARγ and IRAK-M, inhibitory kinases of IRAK molecules, strongly inhibit NF-κB pathway activation by inhibiting MyD88 and IRAK1, thus maintaining the immune tolerance of BECs (179). PPARγ expression in cultured human BECs were downregulated in a Th-1-dominant immune environment, which promotes PBC progression. BECs from patients with PBS are more sensitive to LPS stimulation than those from healthy controls. PPARγ activation by 15d-PGJ2 negatively inhibits LPS-induced NF-κB pathway activation (180). Therefore, PPARγ is involved in negative regulation of BECs to maintain immune tolerance.

## Therapeutic Strategies for Immune Disorders

Reduction of ALP is currently considered an adequate response to treatment and an endpoint in clinical trials. The mechanism of damage by the immune system in PBC, a disease with strong autoimmune characteristics, has not yet been fully elucidated. Drugs with broad immunosuppressive effects, including glucocorticoids (181), cyclosporine (182), and azathioprine (183), have not produced visible beneficial effects on the clinical outcomes in patients with PBC. In addition, selective depletion of B cells with anti-CD20 monoclonal antibody significantly reduced the titer of autoantibodies in patients with PBC, but the therapeutic effect was nonsignificant (135). Other immunomodulators under development include the IL12/23 monoclonal antibody (ustekinumab), CD40/CD40L antagonist, CX3CL1 antibody, CD80/CD86 antagonist, and selective sphingosine-1-phosphate receptor modulator (184). Current animal models cannot fully reproduce the clinical features and immunological complexity of human PBC (185), which makes it difficult to select suitable models. In addition, the lack of immune-related biomarkers for predicting PBC progression and prognosis also complicates PBC research.

**Figure 1:**
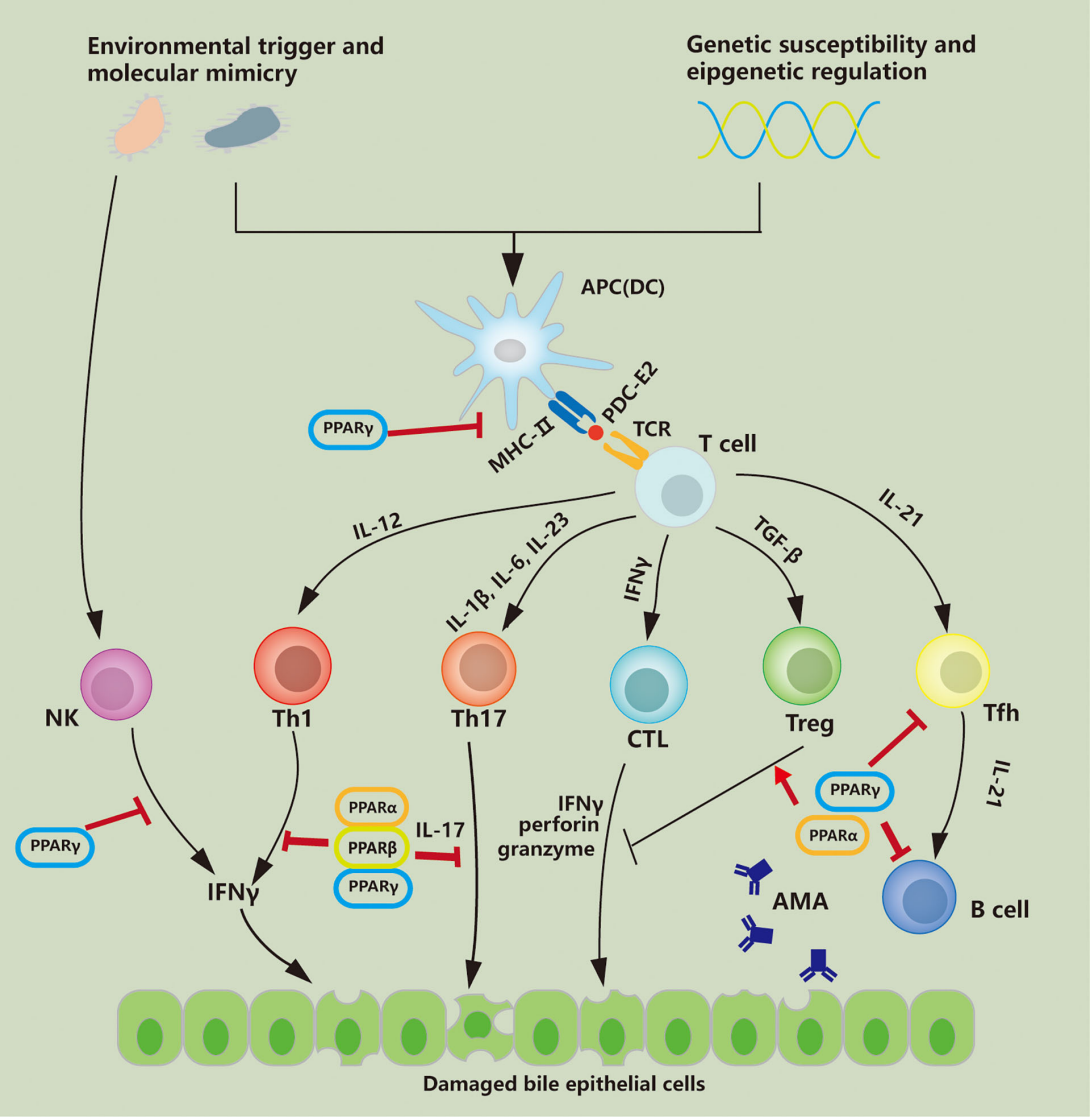
PPAR regulates immune cells involved in PBC pathology. PDC-E2 the E2 component of the mitochondrial pyruvate dehydrogenase complex; APC antigen-presenting cell; DC dendritic cell; IFNγ interferon-γ, TFh follicular helper T cell; AMA anti-mitochondrial autoantibody; TGFβ transforming growth factor-β; Treg regulatory T cell; CTL cytotoxic T lymphocyte; NK natural killer; MHC-II major histocompatibility complex-II; TCR T cell receptor; Th T helper.

The efficacy of PPAR agonists in the treatment of PBC has been confirmed. Previous studies have primarily focused on the regulation of PPARs in bile acid metabolism. It has been proven that regulation of cytochrome P450 enzymes and bile acid transporters by PPARα contributes to hepatic lipid and bile acid homeostasis, which is involved in alleviating cholestatic liver injury (186). Regulation of immune response and inhibition of disease progression by PPARs have been confirmed in studies of other autoimmune diseases, including colitis (187) and autoimmune encephalomyelitis (188). Although studies on PPAR-regulating immunity in PBC are relatively limited, current research results have preliminarily confirmed that activation of PPARs is involved in the reverse of Th1-dominant immune injury, which may delay the progression of PBC. As shown in **Figure 1**, PPARs have regulatory effects on multiple immune cells involved in immune disorders. In **Table 2**, we describe the regulatory effects of different PPAR subtypes on diverse immune cells. In general, PPAR activation promotes the maintenance of immune tolerance by directly or indirectly influencing the differentiation of Th cells. Whether the alterations in immunity are directly related to the decrease in serological indicators or are beneficial to the long-term clinical outcomes of PBC patients requires further evaluation.

**Table 2:** Regulatory effects of different PPAR subtypes on diverse immune cells.

	Abnormality in PBC	PPARα	PPARβ/δ	PPARγ
**Innate immune cells**				
**Monocyte** **/Macrophage**	Hepatic monocytes and macrophages accumulation increase with more proinflammatory cytokines.	PPARα activation promotes macrophage polarization from M1 to M2.	PPARβ/δ activation promotes M2 macrophage polarization.	PPARγ activation inhibits monocyte/macrophage accumulation and promotes M2 macrophage polarization.
**Dendritic cell** **(DC cell)**	Myeloid dendritic cells infiltration increases and inhibit Th2-dominant immune response.	Evidence absence	Evidence absence	PPARγ activation increases self-tolerance of dendritic cells and indirectly inhibits Th1 differentiation from naïve T cells by reduction IL-12 production of dendritic cells.
**Natural killer cell** **(NK cell)**	Frequency of natural killer cells increases with increased IFNγ production.	Evidences absence	Evidences absence	PPARγ activation reduces IFNγ production of NK cells
**Natural killer T cell** **(NKT cell)**	Activated NKT cells aggravates bile epithelial cells damage and promotes primary biliary cholangitis progression.	PPARα activation negatively regulates *ifng* gene transcription.	Evidences absence	PPARγ activation indirectly enhances invariant NKT cell expansion *via* upregulation of CD1d expression in DCs.
**Adaptive immune cells**				
**T helper cells**	Th1 and Th17 dominant immune response, with increased production of IFNγ and IL-17.	Expression of PPARα of CD4^+^ T in male is higher than that in female. PPARα activation inhibits Th1 and Th17 differentiation.	PPARδ activation inhibits IFNγ and IL-17 production.	PPARγ activation promotes Th1 phenotypic conversion to Th2 and inhibits Th17 polarization.
**Follicular helper** **T cell** **(Tfh cell)**	Frequency of CD4+CXCR5+ Tfh cells increases in PBC patients. Reduction of Tfh cells indicates adequate response to UDCA treatment.	Evidences absence.	Evidences absence.	PPARγ agonist inhibits Tfh cell response.
**Regulatory T cells** **(Treg cell)**	Relative number of CD4^+^CD25^+^ Treg cells and Foxp3 expressing Tregs reduce in PBC patients.	PPARα agonist promotes Foxp3^+^regulatory T cells differentiation.	Evidences absence	PPARγ agonist promotes Foxp3 expression and increases hepatic CD4^+^CD25^+^Foxp3^+^ Treg cells percentage.
**B cell**	IgM-producing plasma cells increases	Bezafibrate inhibits B cell maturation by down-regulation of B cell activating factor.	Evidences absence	Down-regulation of PPARγ is responsible for proliferation and antigen-specific immune response of B cells.
**Bile epithelial cell** **(BEC)**	Toll like receptor 4 in BECs recognize pathogen-associated molecular patterns in bile and NF-κB and MAPK pathways are activated subsequently.	Evidences absence	Evidences absence	PPARγ activation inhibits NF-κB pathway and maintain immune tolerance of BECs to pathogen-associated molecular patterns.

## Summary and Prospects

The new generation of farnesoid X receptor and PPAR agonists and bile acid uptake inhibitors have effectively expanded the second-line treatment of PBC. UDCA still occupies a dominant position in the treatment of PBC, with its incomparable safety and effectiveness, as confirmed by several clinical trials. Fibrates are currently included in the clinical guidelines for add-on therapy (189). It is not known whether PPAR agonists will be used as monotherapy in the future or in combination with UDCA in patients with PBC, regardless of adequate response to UDCA. PPARα, PPARβ/δ, and PPARγ have distinct, but complementary functions. Dual- or pan-PPAR agonists may have better therapeutic effects than selective agonists. Activation of different subtypes of PPARs has beneficial effects on upstream immune disorders, midstream cholestasis (186), and downstream fibrosis (190) of PBC progression. The side effects may be a barrier to the application of PPAR agonists. Increased creatinine levels and myalgia are common side effects of PBC treatment (29). Cardiotoxicity, hepatotoxicity, and tumorigenesis of PPAR activation also indicate that PPAR agonists should be used circumspectly (191). Agonists with stronger liver targeting and more balanced activation effects may be more competitive in the future.

In this review, we comprehensively summarize the regulation of PPARs on known immune abnormalities of PBC. However, the full picture of the pathogenesis of PBC is not yet understood. In addition, not all the immune cells involved in PBC pathogenesis are associated with PPARs, such as cytotoxic T cells, although regulatory effects have been demonstrated in anti-tumor researches. Therefore, with the deepening of understanding about PBC immunopathogenesis, the regulatory roles of PPARs will be further updated. Interestingly, the expression of PPARα in T cells has gender differences, and whether this difference is related to the female dominance of PBC should be further explored. Comparison of PBC patients who have adequate response to UDCA in combination with fibrates therapy but not to UDCA monotherapy may obtain novel biomarkers which could predict disease progression and treatment response, such as sCD163. Although animal models of PBC are still defective, the effects of PPARs on immune cells in current autoimmunity mice model, such as dnTGF-βRII and IL-2Rα^-/-^ mice models (185), are worthy of further exploration.

## Author Contributions

CW and YS completed manuscript writing. XW and HM helped to search literature. QL provided guidance on writing. YG and JN are jointly responsible for the structure of this manuscript. All authors contributed to the article and approved the submitted version.

## Conflict of Interest

The authors declare that the research was conducted in the absence of any commercial or financial relationships that could be construed as a potential conflict of interest.

## Publisher’s Note

All claims expressed in this article are solely those of the authors and do not necessarily represent those of their affiliated organizations, or those of the publisher, the editors and the reviewers. Any product that may be evaluated in this article, or claim that may be made by its manufacturer, is not guaranteed or endorsed by the publisher.
